# Ginkgo Seed as Medicine–Food Homology for Migraine: Network Pharmacology and Molecular Docking Insights

**DOI:** 10.3390/ijms26189225

**Published:** 2025-09-21

**Authors:** Zhifan Li, Shuangyuan Yu, Bolin Chen, Erzheng Su, Fuliang Cao

**Affiliations:** 1 Co-Innovation Center for the Sustainable Forestry in Southern China, Nanjing Forestry University, Nanjing 210037, China; lizhifanfan@163.com (Z.L.); ysy199949@163.com (S.Y.); ezhsu@njfu.edu.cn (E.S.); 2 Department of Food Science and Technology, College of Light Industry and Food Engineering, Nanjing Forestry University, Nanjing 210037, China; 3 Hunan Academy of Forestry Sciences, Changsha 414000, China; cbl92@njfu.edu.cn; 4 The Jiangsu Provincial Platform for Conservation and Utilazation of Agricultural Germplasm, Nanjing 210014, China

**Keywords:** migraine, ginkgo seed, network pharmacology, molecular docking, mechanism

## Abstract

As a medicinal and edible homologous substance, ginkgo seeds’ historical application in headache management, documented from Diannan Materia Medica to contemporary clinical practice, is based on empirical evidence. This study employed network pharmacology and molecular docking to explore the anti-migraine mechanisms of ginkgo seeds. In total, 10 related signal pathways (cancer pathway, lipid and atherosclerosis, *Phosphatidylinositol 3-Kinase–Protein Kinase B* (*PI3K-AKT*) signaling pathway, etc.) and 10 hub genes were identified through Gene Ontology (GO) functional annotation, Kyoto Encyclopedia of Genes and Genomes (KEGG) pathway enrichment, and in the inprotein–protein interaction (PPI) network. Molecular docking results demonstrated that formononetin, stigmasterol, and beta-sitosterol in ginkgo seeds can interact with 10 core targets (average binding energy ≤ −3.17 kcal/mol). This study analyzed the related pathways and targets of ginkgo seeds in the treatment of migraines, as well as the docking test of small-molecule ligands and target protein receptors, which provides a reference with which to find and explore effective preventive health foods for migraines.

## 1. Introduction

Migraine is a common chronic neurovascular disease characterized by recurrent pulsatile or paroxysmal headaches and accompanied by autonomic symptoms such as arrhythmia, nausea and vomiting, photophobia, and phonophobia [1]. The World Health Organization (WHO) ranks migraine as the third most common disease and the sixth most disabling neurological disorder in the world [2,3]. Approximately 15% of the global population suffers from migraines, including more women (33%) than men (18%), and an annual incidence of more than 10%, resulting in a significant disability burden for both individuals and society [4,5]. However, migraine is a multifactor disease, and its pathophysiology involves genetic changes in brain excitability, intracranial artery dilation, cortical spreading depression, sensitization of the trigeminal vascular pathway, and regulation of the microbiome–gut–brain axis [6,7]. This also leads to the fact that commonly used therapeutic drugs are mainly focused on improving symptoms and acute attacks, with limitations in preventing recurrence and long-term efficacy, and susceptibility to drug resistance and even serious adverse reactions [8,9]. Therefore, the exploration of medicinal and edible homologous substances that can be taken for a long time with milder symptoms and better toleration rates has become a promising approach for the prevention and treatment of migraines [10,11,12].

In recent years, the effect of traditional Chinese medicine (TCM) has been investigated as an intervention with remarkable results [13,14]. Looking back at the ancient classical prescriptions, the “San Pian Tang” recorded in the “Bian Zheng Lu” defined migraine as “Tou Feng”, and the “Qing Shang Juan Tong Tang” classified migraine as a headache in the “Shou Shi Bao yuan”. Ginkgo seeds, the kernel of ginkgo biloba L. (a species belonging to the Ginkgoaceae family native to China), contain a variety of bioactive compounds, including flavonoids, terpenoids, alkylsalicylic acid derivatives, polysaccharides, and amino acids. It exhibits multiple bioactivities such as antioxidant, antimicrobial, and insecticidal properties [15] and possesses various pharmacological effects. For instance, Kang proved that ginkgo seeds have potential anti-cardiovascular disease effects due to their hypocholesterolemic activity via the down-regulation of lipid metabolism in mice receiving a high-fat diet [16]. Yu revealed that microwave-heated ginkgo seeds exert a neuroprotective effect, which can significantly improve the learning and memory abilities of Amyloid Precursor Protein/Presenilin-1 (APP/PS1) mice and inhibit apoptosis and degeneration of nerve cells [17]. Shen et al. reported that ginkgo seeds promote longevity and stress resistance of Caenorhabditis elegans [18]. Through collecting and sorting out famous and experienced prescriptions from ancient and modern times, we found that ginkgo seeds are almost a single combination in the treatment of migraine. The Diannan Materia Medica states, “Crushing and applying ginkgo seed flesh to the temples can relieve headaches and eye pain”. The Journal of Traditional Chinese Medicine records that ginkgo seeds can be used to treat neuropathic headaches. Previous study reported that “Bai Guo Chuan Xiong Tang” treated 98 cases of headache, and ginkgo seeds were the preferred medication in this formula, with a cure rate of up to 83%. However, the above phenomenon was only explored as an empirical method, lacking exploration of the anti-migraine mechanism of ginkgo seeds.

Network pharmacology, as a branch of systems biology, is constantly emerging internationally. It excels in systematically revealing the characteristics of the relationship between drugs and diseases from a holistic perspective, making it consistent with the principles of the holistic view and dialectical treatment in TCM [19]. Molecular docking technology is widely used in drug research and development due to its ability to simulate the interaction mode and binding energy between small-molecule ligands and target protein receptors [20]. However, it is important to acknowledge its inherent limitations, such as the simplified treatment of solvent effects, the challenge of accurately modeling the full flexibility of the receptor and ligand, and the potential for false positives or negatives due to the approximations in scoring functions. In addition, the target of action in vivo and the mechanism of action for TCM are numerous and very complex due to its intricate composition, so it is difficult to study their mechanism using animal or cell experiments alone [21,22]. Based on this, this study aims to provide a more comprehensive understanding of the potential anti-migraine mechanisms of ginkgo seeds, which includes screening of disease-related active ingredients, targets, and pathways through network pharmacology, and further validation of the above results using molecular docking technology.

## 2. Results

### 2.1. Screening of Active Compounds and Targets of Ginkgo Seed

A total of 21 active compounds in ginkgo seeds, with attributes of oral bioavailability (OB) ≥ 30% and drug-likeness (DL) ≥ 0.18, were retrieved by the traditional Chinese medicine system pharmacology (TCMSP) database. The basic information of some active compounds is shown in Table 1. Based on the TCMSP database, a total of 580 protein targets related to the above active compounds were obtained, and 291 gene symbols were obtained after gene annotation using the Uniprot database.

### 2.2. Intersection Targets of Ginkgo Seed and Migraine

**Figure 1:**
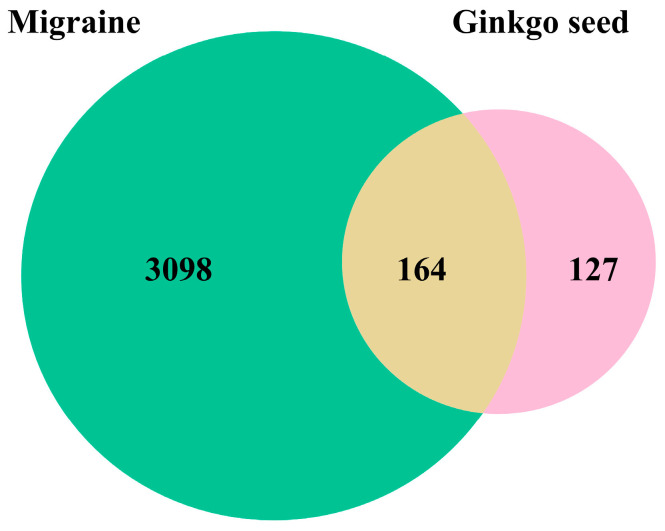
Venn diagram of ginkgo seeds and migraine. The overlapped genes were considered the potential hub genes used by ginkgo seeds against migraine.

A total of 3262 migraine-related target genes were extracted by searching with the keyword “migraine” and integrating the collected targets to retain unique values from GeneCards and Online Mendelian Inheritance in Man (OMIM) databases. Through the Venny 2.1.0 software, 164 genes for ginkgo seeds and migraine were obtained by crossing the traditional Chinese medicine targets with disease genes, as shown in Figure 1.

### 2.3. Construction of “Compound–Target” Network

The interrelation network between 14 active ingredients and 164 genes obtained above was analyzed using Cytoscape 3.9.0 software, which consisted of 178 nodes (164 nodes of gene targets and 14 nodes of active ingredients) and 353 edges, as shown in Figure S1. According to CytoNCA analysis, the active ingredients are ranked in descending order of degree values, as shown in Table 2. Among them, quercetin, (-)-epigallocatechin-3-gallate, kaempferol, β-sitosterol, isorhamnetin, formononetin, and stigmasterol are the key pharmacological molecules of ginkgo seeds in the treatment of migraine due to their high degree values, spacing centrality, and tight centrality. Under the conditions of degree ≥ 3, betweenness ≥ 99.86, and closeness ≥ 0.35, 164 target genes in the network diagram were screened and sorted in descending order of degree values, as shown in Table 3. *PTGS2*, *PTGS1*, *PPARG*, *ADRB2*, *GABRA1*, and *NOS3* are potential targets for the treatment of migraines using ginkgo seeds due to their advantageous values.

### 2.4. PPI Network Analysis

A total of 164 gene targets were introduced into String to construct an inprotein–protein interaction network (PPI), and the isolated nodes were removed, as shown in Figure S2. A total of 163 nodes and 8212 edges had an average degree value of 50.1 in the PPI network. The proteins in the PPI network are represented by circular nodes with a 3D protein structure inside, and the lines between the nodes represent the interactions between proteins. The more lines there are, the stronger the relationship between them. To further explore the core targets, the results document from the String database was imported into Cytoscape 3.9.1 software, and the CytoNCA plug-in was used to filter by selecting data with degrees, betweenness, presence, eigenvector, Local Attachment Coefficient (LAC), and network all greater than the median value. Based on the calculation results, the first screening was carried out under the conditions of degree ≥ 94, betweenness ≥ 50.03, closeness ≥ 0.56, eigenvector ≥ 0.07, LAC ≥ 73.33, and network ≥ 76.11. As a result, 62 targets were obtained with 3370 interrelationships between them, and a subnetwork was constructed within the PPI network, which was further calculated using the CytoNCA plug-in. In total, 29 core targets were obtained with 812 interrelationships among the targets after the second screening was carried out under the conditions of degree ≥ 112, betweenness ≥ 6.85, closeness ≥ 0.92, eigenvector ≥ 0.13, LAC ≥ 100.53, and network ≥ 106.78. The first 10 targets were *TNF*, *AKT1*, *IL6*, *MMP9*, *PTGS2*, *BCL2*, *ESR1*, *CASP3*, *ALB*, and *PPARG*, which play a crucial role in PPI, and the screening process is shown in Figure S2.

### 2.5. GO and KEGG Pathways Enrichment Analysis

**Figure 2:**
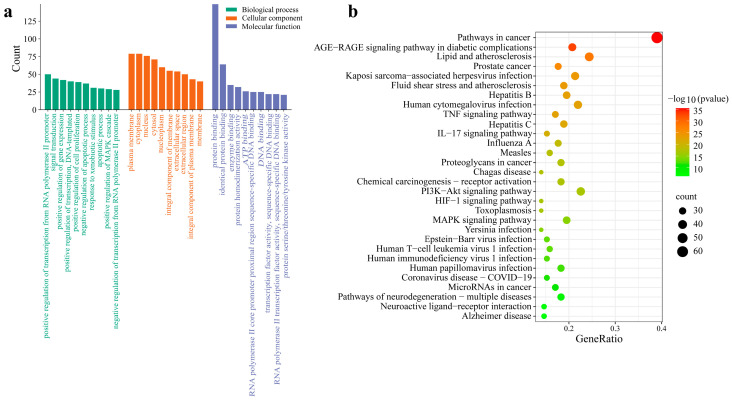
The 10 most significant signaling pathways of Gene Ontology functional analysis (**a**) and the first 30 from the Kyoto Encyclopedia of Genes and Genomes pathway enrichment analysis (**b**) for therapy target genes of ginkgo seeds on migraines.

To further understand the functions of core targets and the pathways of the action of active compounds, we performed Gene Ontology (GO) and Kyoto Encyclopedia of Genes and Genomes (KEGG) pathway enrichment analysis using the DAVID database and visualized the results through the Microbiology Letter Platform. A total of 940 GO items were obtained (*p* < 0.01), among which 731 items of biological process (BP) were closely related to the positive regulation of RNA polymerase II promoter transcription, signal transduction, gene expression, transcription, DNA template, etc. The 81 items of cell composition (CC) are mainly related to the plasma membrane, cytoplasm, nucleus, cytosol, and so on. The 128 results of molecular function (MF) are mainly related to protein binding, identical protein binding, enzyme binding, protein homologous dimerization activity, etc. The top 10 results were selected, respectively, as shown in Figure 2a. The longer the bar chart, the more targets the item contains.

In order to comprehensively study the role of ginkgo seeds in the prevention and treatment of migraines, 182 pathways were screened by KEGG pathway enrichment analysis (*p* < 0.01). The five signaling pathways with the largest number of targets were cancer pathogenesis, lipids atherosclerosis, the PI3K-Akt signaling pathway, human cytomegalovirus infection, and Kaposi sarcoma-associated herpesvirus infection, with 64, 40, 37, 36, and 35 enrichment targets, respectively, suggesting that ginkgo seeds may play a therapeutic role in migraines through these pathways. The first 30 signaling pathways with a smaller *p* value and more counts of corresponding target protein are shown in Figure 2b, where the size and color of the bubble indicate counts and *p* values, respectively. The darker the color, the smaller the *p* value.

### 2.6. Construction of Bioactive Compound-Target-Pathway Network

**Figure 3:**
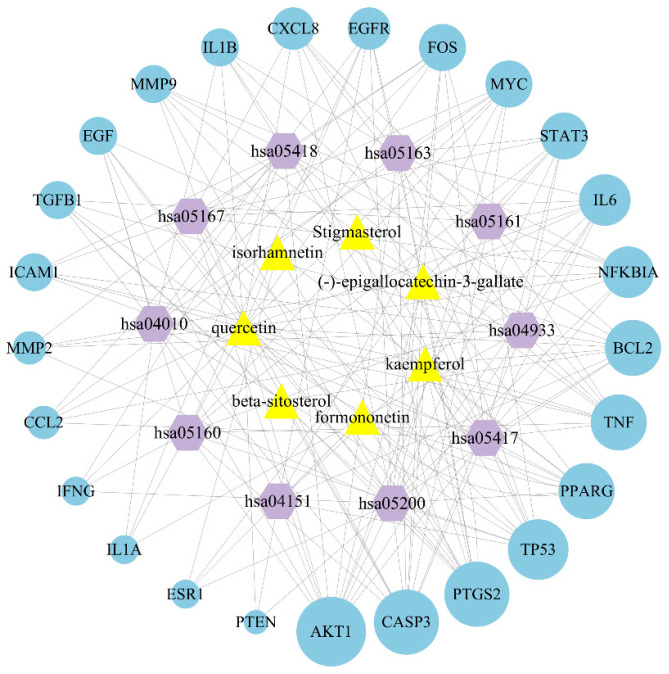
The network of “Bioactive Compound–Target–Pathway” for ginkgo seeds in the treatment of migraines. The blue nodes represent targets; the purple nodes represent pathways; and the yellow nodes represent active ingredients. The edges represent the interactions between them, and the node size is proportional to their degree.

An “Bioactive Compound–Target–Pathway” network was constructed using Cytoscape3.9.0 (Figure 3), in which nodes of different colors and shapes represent different bioactive compounds, targets, and pathways, including 7 compounds (yellow triangle), 29 targets (blue circle) and 10 KEGG pathways (purple hexagon), and the number of connections between nodes represents the importance of nodes in the network. The node size represents the node scores obtained using the cytoNCA plug-in in Cytoscape3.9.0.

### 2.7. Molecular Docking

The targets of seven key pharmacophore molecules were seen in the 2.3 intersect, with 10 target proteins obtained in the 2.4 intersect. After ranking the number of key pharmacophore molecules corresponding to the core targets from high to low, *PTGS2*, *PPARG*, *BCL2*, *CASP3*, *TNF*, *AKT1*, and *ESR1* were screened as the core proteins that matched the most pharmacophore molecules. The structure of these proteins was searched in the PDB database and used as receptor proteins to perform molecular docking with their corresponding pharmacodynamic molecules using Auto-DockTools software. The docking results are shown in Table S1. When the binding energy value is <0, the small-molecule ligand and docking receptor protein can spontaneously bind in a natural state, and can even be integrated under the condition of a binding energy value < −1.2. The degree of conformational stability between the small-molecule ligand and docking receptor protein is inversely proportional to the binding energy size [23].

**Figure 4:**
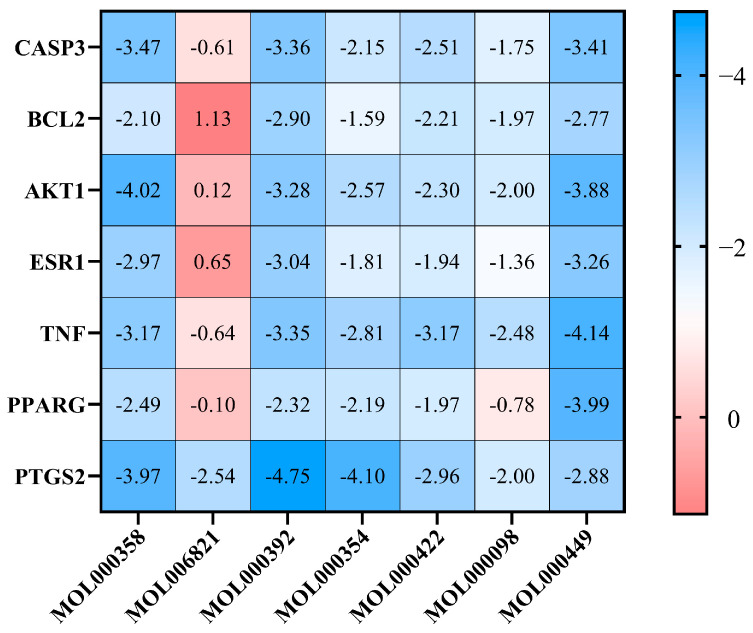
Thermal map of binding energy (kcal/mol) between active ingredients and target proteins for ginkgo seeds in the treatment of migraines. The *y*-axis represents the name of targets; the *x*-axis represents the MOL ID of effective ingredients; and the blue intensity is directly proportional to the absolute value of the binding energy.

**Figure 5:**
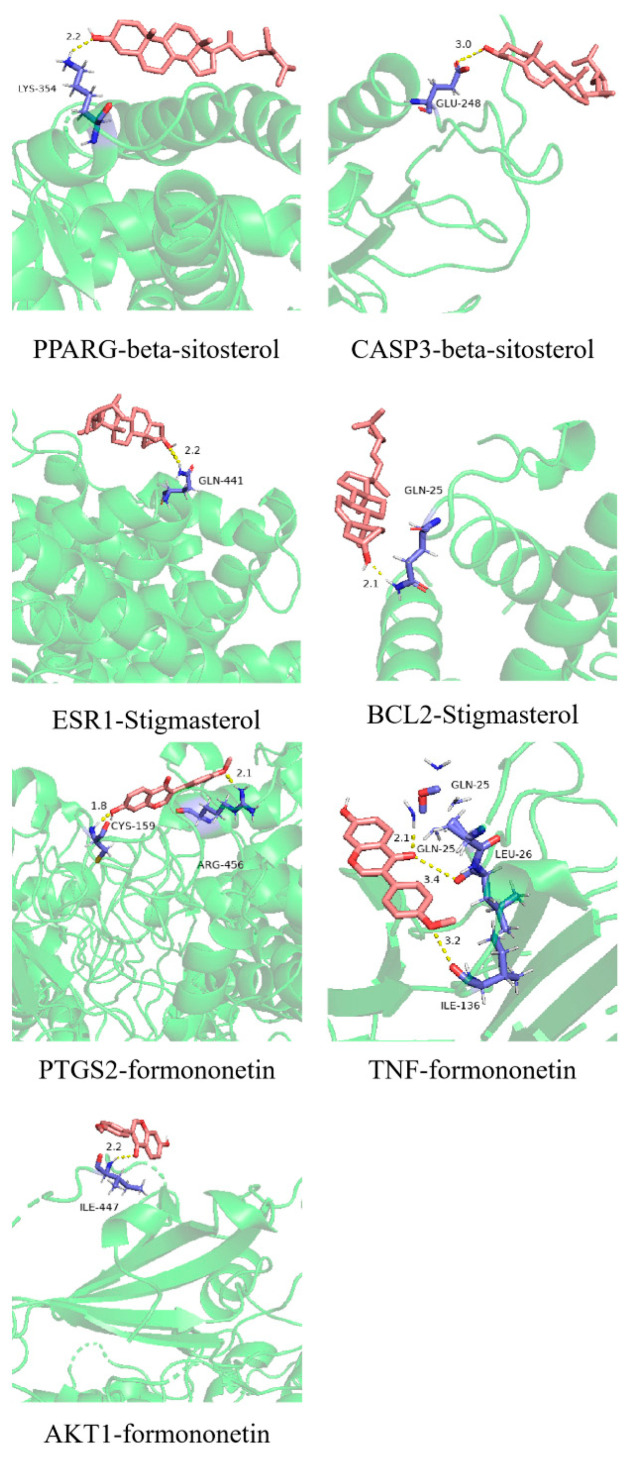
Optimal ligand–receptor molecular docking sites in ginkgo seed therapy for migraines: structural representation of ligands (red), receptor protein (green) and their binding groups on receptor proteins (blue). (Ligands: beta-sitosterol, stigmasterol, formononetin; Receptors: *PPARG*, *CASP3*, *ESR1*, *BCL2*, *PTGS2*, *TNF*, *AKT1*).

As shown in Figure 4, the docking scores of pharmacophore molecules such as formononetin, β-sitosterol, and stigmasterol were higher overall (average binding energy ≤ −3.17 kcal/mol), which suggests that they could be the core pharmacophore molecules in ginkgo seeds that can treat migraines. *PTGS2* had the largest docking number of pharmacophore molecules and relatively high scores, followed by *AKT1* and *TNF*. Next, Pymol software was used to visualize the optimal conformation of the docking of 7 proteins, and the specific binding site is shown in Figure 5. The active ingredients of ginkgo seeds were closely bound to the target proteins by hydrogen bonding and van der Waals forces, in which β-sitosterol was bound to lysine 354 residues of the *PPARG* protein and glutamate 248 residues of the *CASP3* protein by hydrogen bonding. Stigmasterol was bound to the glutamine 441 residue of the *ESR1* protein and glutamine 25 residues of the *BCL2* protein by hydrogen bonding. Formononetin was bound to cysteine 159 and arginine 456 residues of the *PTGS2* protein; glutamine 25 and isoleucine 26 residues of the *TNF* protein, and isoleucine 447 residue of the *AKT1* protein were bound via hydrogen bonding.

## 3. Discussion

The core components of ginkgo seeds, including quercetin, (-)-epigallocatechin-3-gallate, kaempferol, beta-sitosterol, isorhamnetin, formononetin, and stigmasterol, play important roles in the interaction network and have good binding ability with targets. Quercitrin is a naturally available type of flavonoid with a wide spectrum of bioactivities, including antioxidative stress, anti-inflammation, anti-microorganisms, immunomodulation, analgesia, wound healing, and vasodilation [24]. The inhibition of prostaglandin production, neuroinflammation, and oxidative stress revealed its significant analgesic and anti-inflammatory effects against chronic pain [25,26]. Previous studies have shown that quercetin can dose-dependently (5, 10, 15 mg/kg) improve pain behavior in a rat model of migraines induced by nitroglycerin (NTG) through its inhibition of nitric oxide, proinflammatory cytokines, *CGRP*, and immunoreactive C-fos cells, which are involved in the development of migraines [27]. In addition, quercetin reduces the production of IL-1β-induced prostaglandin E2 (PGE2), which can trigger migraines [28,29]. In the cerebral cortex and hippocampus of mice, quercetin can protect the mitochondrial apoptotic pathway and prevent neuronal degeneration by regulating *BAX/BCL2*, reducing activated cytochrome c, *CASP3* activities, and lysing *PARP-1*, which also has a neuroprotective effect on adult mice adversely affected by Lipopolysaccharide (LPS) [30]. (-)-epigallocatechin-3-gallate has clearing radical, antioxidant, anti-inflammatory, and anti-apoptotic characteristics, which can cross the blood–brain barrier and reach nerve tissue even at low concentrations to achieve neuroprotective and anti-inflammatory properties that accompany pain improvement in the brain and nerve damage [31,32]. β-sitosterol and stigmasterol are common classes of plant sterols and components of various TCM plants, which have various biological activities such as antioxidant, anti-inflammatory, and anti-apoptotic traits [33]. Studies have shown that taking β-sitosterol as a dietary supplement inhibits the activation of trigeminal neurons and may be beneficial in the treatment of migraines [34]. β-Sitosterol treatment (10 mg/kg) also has anxiolytic effects on migraines, which can be demonstrated through ameliorating oxidative/nitrosative stress and enhancing mitochondrial function [35]. In animal models of Alzheimer’s disease, plant sterols (β-sitosterol and stigmasterol) improve cognitive function by reducing acetylcholinesterase or acetylcholinesterase (AChE/BChE) activity and free radical load in the cerebral cortex and hippocampus [36]. Formononetin, a non-steroidal isoflavone compound, is a bioactive component of many medicinal plants and has excellent effects, such as anti-inflammatory, antioxidant, and anticancer properties [37]. For instance, formononetin can reduce neuroinflammation through related signaling pathways, protect neurons from oxidative stress and toxicity induced by L-glutamate or amyloid beta protein, and increase *estrogen receptor β(ER-β)* protein expression [38]. Ginkgolide B, a component not involved in the network, but which can regulate glutaminergic transmission and anti-platelet activator (PAF) in the central nervous system, is considered by researchers as a promising pharmacological adjunct for the treatment of adult migraines [39]. One complex, ginkgolide B/coenzyme Q10/riboflavin/magnesium, when orally administered during prophylactic therapy, can reduce the disabling aspect of migraines in childhood [40]. However, previous studies have almost exclusively used complexes extracted from ginkgo biloba leaves, and ginkgolide B derived from ginkgo seeds has not received much attention due to its lower content. Although kaempferol, isorhamnetin, and formononetin have anti-inflammatory, antioxidant, antitumor, and immune-regulating activities, few studies have been conducted on their effects on migraines, which may be a new direction for the study of TCM active ingredients in the future [41,42,43].

In this study, 10 targets, including *PTGS2*, *PPARG*, *BCL2*, *CASP3*, *TNF*, *AKT1*, and *ESR1*, were selected through PPI topological analysis using network pharmacology. *PTGS2* is a prostaglandin peroxidase synthetase 2, which is a key enzyme in the conversion of arachidonic acid to prostaglandin [44]. Under mechanical, chemical, and physical stimuli, *PTGS2* is overexpressed and plays a critical role in the promotion and development of inflammatory response [45]. Studies have shown that *PTGS2* participates in the pathogenesis of migraines, and the symptoms of migraine patients can be improved by inhibiting the *PTGS2* proinflammatory pathway and reducing the levels of *TNF-α*, *IL-2*, and *IL-6* [46]. *PPARG* is a peroxisome proliferator-activated receptor γ, which has neuroprotective potential for its relationship with neuroinflammation and neurodegeneration [47,48]. *BCL2* is B-cell lymphoma 2, which is one of the ways to study the mechanism of action related to the anti-neuroinflammation of drugs. Research found that the compounds of methanol extract and selenium nanoparticles of hairy tengma leaves can counteract nerve loss by increasing levels of *BCL2* and brain-derived neurotrophic factors [49]. The neuroprotective effect of astaxanthin on the retina can be evaluated by the mRNA and protein levels of *BAX* and *BCL2* in retinal ganglion cells [50]. The mechanism of the effect of stavudine on inflammation in Alzheimer’s cells has been studied by detecting the expression of proteins such as *BCL2* and *CASP3* in Western blot [51]. *CASP3* is cysteine protease 3, which can be activated by Reactive Oxygen Species (ROS) to cause apoptosis, especially in brain diseases [52]. Neurogenic neuroinflammation plays a wide-ranging role in the pathogenesis of migraines, including the transition from episodic migraines to chronic migraines [53]. High levels of *TNF-α* have the inherent ability to enhance damage related to neurological diseases, which takes part in the body’s immune regulation and inflammatory response, so inhibiting its signal transduction may be an effective strategy for treating migraines [54,55]. *AKT1*, a serine/threonine kinase, is a multifunctional protein involved in the regulation of cell growth, survival, and proliferation [56]. Lin et al. confirmed that puerarin exerts anti-neuroinflammatory effects on sepsis-related encephalopathy by regulating the *AKT1* pathway in microglia during in vivo and in vitro experiments [57]. *ESR1* is an estrogen receptor present in many areas of the brain, including the hippocampus, cortex, amygdala, and striatum. The binding of estrogen with estrogen receptors can exert genomic or non-genomic effects through interactions with extracellular or nuclear estrogen receptors and plays a variety of different roles to promote neuroprotection [58]. In addition, the phenomenon that women have a higher prevalence of migraines is related to estrogen levels [59].

Molecular docking methods further verified the binding ability of the above components and target proteins. The results of molecular docking binding energy showed that the core components of ginkgo seeds could bind well to the seven targets in their natural state, except (-)-epigallocatechin-3-gallate, which provides a new line of inquiry for experiments to verify the treatment of migraine by ginkgo seeds. Among them, the binding energy of the active ingredients with *PTGS2* can reach up to −4.75 kcal/mol, indicating that the active ingredients of ginkgo seeds can treat migraine by down-regulating the protein expression of *PTGS2*, and then blocking the release of prostaglandins such as *PGE2.* Through the analysis of the KEGG pathway, it was found that cancer, lipid and atherosclerosis, and *PI3K-Akt* signaling pathways are the main treatment regimens. Migraine is associated with vascular events such as stroke and cardiovascular diseases, which are well-known to have a close connection with arterial atherosclerosis. Migraine sufferers are more prone to developing arterial atherosclerosis, which contains eleven targets including *Akt1, TNF, IL-6*, etc. [60]. Furthermore, the *PI3K-Akt* signaling pathway is an important survival signal transduction pathway in cells; when activated in the brain tissue of a rat migraine model, it involves seven targets, including *Akt1, IL-6,* and *BCL2* [61]. The above pathways involve the *Akt1* target. Studies found that *Akt1* is extensively involved in the regulation of microglia-mediated neuroinflammation, which leads to central sensitization and thereby triggers migraines [62,63,64,65]. San Pian decoction can treat migraines by preventing inflammation through regulating inflammatory cytokines and key genes and proteins in the *PI3K/AKT* and *MAPK* signaling pathways [66]. Ginkgo seeds could exert anti-neuroinflammatory effects by acting on receptor proteins such as *Akt1* through the lipid and atherosclerosis and *PI3K-Akt* signaling pathways, reducing the phosphorylation level of *Akt1*, and thereby delaying the process of central sensitization and alleviating migraine symptoms.

In the future, we will focus on experimentally validating the predicted mechanisms by building on the theoretical framework established in this study through quantitative PCR and Western blot analysis in animal models of migraines. These experiments will help clarify the precise roles of key candidate targets and determine their functional relevance in a physiological context. We anticipate that these follow-up studies will not only validate our computational predictions but also provide deeper mechanistic insights, potentially facilitating the development of ginkgo seed-based interventions for migraine prophylaxis and treatment.

## 4. Materials and Methods

### 4.1. Screening Strategy for Active Components and Targets in Ginkgo Seeds

All components of ginkgo seeds were retrieved from the TCMSP database (https://www.tcmsp-e.com (accessed on 15 April 2025), version 2.3, updated on 27 April 2024) [67]. To ensure effective oral activity, TCM must surmount the challenges associated with absorption distribution, metabolism, and excretion (ADME) processes, which are related to OB and DL. OB is one of the most significant pharmacokinetic parameters, and substances with high OB values (OB ≥ 30%) are usually determined as active components. DL, a qualitative concept applied in drug design to estimate the druggability of a molecule, is conducive to rapid screening of active substances. Substances with a DL index ≥ 0.18 are considered to have high medicinal properties. Therefore, the compounds in ginkgo seeds with OB ≥ 30% and a DL index ≥ 0.18 were deemed to be pharmacokinetically active in the current study, and the targets of active ingredients were collected from the TCMSP database. Subsequently, gene annotation was performed using the UniProt database (http://www.uniprot.org/ (accessed on 30 April 2025), updated on 23 April 2025), and the repeated targets were eliminated after merging [68].

### 4.2. Prediction of Related Targets of Ginkgo Seed and Migraine

Using GeneCards (https://www.genecards.org/ (accessed on 3 May 2025), version 5.24, updated on 1 April 2025) and online mendelian inheritance in man (OMIM) (https://omim.org/ (accessed on 3 May 2025), updated on April 2025) database searches for related genes were conducted through the keyword “migraine” and duplicates were removed [69,70]. Subsequently, the target of active ingredients in ginkgo seeds obtained above was intersected with migraine-related genes using Venny 2.1.0 software.

### 4.3. Draw “Compound-Target” Network

The information on active ingredients and gene targets of intersection between ginkgo seeds and migraine was imported into Cytoscape3.9.0 software (version 3.9.0, University of California San Diego, Santiago, the United States) to construct the relationship network diagram of “compound–target” for visual analysis [71].

### 4.4. Protein–Protein Interaction (PPI) Network

Multiple proteins from intersection target genes were introduced into String (https://string-db.org/ (accessed on 15 May 2025), version 12.0, updated on 26 July 2023), and the species was selected as “Homo sapiens” to predict the potential associated PPI network [72]. Downloads were placed in the inprotein–protein interaction TSV file and imported into Cytoscape 3.9.0 software to screen for core target proteins using the CytoNCA plug-in.

### 4.5. GO and KEGG Enrichment Analysis

The gene Ontology (GO) and Kyoto Encyclopedia of Genes and Genomes (KEGG) enrichment analyses were conducted using the functional annotation tool of the DAVID bioinformatics resources (https://davidbioinformatics.nih.gov/ (accessed on 20 May 2025), updated on 17 April 2025) database [73,74,75]. *p* < 0.05 was selected for functional annotation clustering and as a significant pathway for cluster analysis. Subsequently, the microbiology letter platform (http://www.bioinformatics.com.cn/ (accessed on 21 May 2025)) was used to visualize the top 20 GO items and top 30 KEGG items screened according to the enrichment degree through using bar charts and bubble charts, respectively.

### 4.6. Constructing Ginkgo Seed–Target–Pathway Network

Leveraging the data procured from the aforementioned procedures, Cytoscape 3.9.0 software was employed to construct a comprehensive network elucidating the intricate connections among active compounds, potential targets, and pertinent KEGG pathways, thereby facilitating a more intuitive comprehension of the interplay between each node within the compound–target–pathway network.

### 4.7. Molecular Docking

The mol2 format structures of active compounds were downloaded from the TCMSP database, which were saved as ligand parameter files in pdbqt format by AutoDockTools-1.5.6 software (version 1.5.6, Molecular Graphics Laboratory, Santiago, CA, USA) [76]. The 3D structures of the core target proteins were downloaded from the PDB (http://www.rcsb.org/ (accessed on 25 May 2025), updated on 14 January 2024) database, and were saved in pdbqt format by hydrogenating and calculating their solvent and water molecules using AutoDockTools-1.5.6 software. Subsequently, the appropriate box center and box lattice parameters were set, which included the active pocket sites that small molecular ligands could bind to. Molecular docking was carried out, and 3D diagrams were drawn using PyMol software (Version 3.1, Schrödinger, Inc., New York, NY, USA) for the docking results with the strongest binding activity of each target protein [77]. The binding energy heat map was plotted using Origin software (Version 2021, OriginLab, Northampton, MA, USA).

## 5. Conclusions

In this study, the active components of ginkgo seeds, potential targets, and key pathways acting on migraines were identified by the methods of network pharmacology and molecular docking, which once again confirms the process of multicomponent, multitarget, and multi-pathway treatment of diseases with TCM. Active components such as beta-sitosterol, stigmasterol, and formononetin may regulate levels of phosphorylation such as Akt1 through the lipid and atherosclerosis and PI3K-Akt signaling pathways. Moreover, molecular docking results demonstrated that these core components have a strong affinity with multiple target proteins such as PTGS2, PPARG, BCL2, CASP3, TNF, and ESR1, suggesting that ginkgo seeds exert their therapeutic effects on migraines through multiple targets and pathways. Due to the limitations of the database and the software itself, the above results require further molecular dynamics simulations and in vivo and in vitro experiments for confirmation.

## Figures and Tables

**Table 1:** The information table of active compounds of ginkgo seeds.

MOL ID	Molecule Name	MW	OB (%)	DL
MOL011072	Quinicine	324.46	75.44	0.33
MOL011074	Scillaren A_qt	384.56	57.67	0.78
MOL011075	Shikodonin	362.46	78.16	0.56
MOL001771	poriferast-5-en-3beta-ol	414.79	36.91	0.75
MOL002773	beta-carotene	536.96	37.18	0.58
MOL000449	Stigmasterol	412.77	43.83	0.76
MOL000354	isorhamnetin	316.28	49.6	0.31
MOL000358	beta-sitosterol	414.79	36.91	0.75
MOL000392	formononetin	268.28	69.67	0.21
MOL000422	kaempferol	286.25	41.88	0.24
MOL004350	Ruvoside_qt	390.57	36.12	0.76
MOL000492	(+)-catechin	290.29	54.83	0.24
MOL005236	gibberellin	346.41	81.59	0.53
MOL006821	(-)-epigallocatechin-3-gallate	458.4	55.09	0.77
MOL000098	quercetin	302.25	46.43	0.28

**Table 2:** Information table of effective ingredients in the treatment of migraines with ginkgo seeds.

MOL ID	Molecule Name	Degree	Betweenness	Closeness
MOL000098	quercetin	99	17,269.80	0.53
MOL006821	(-)-epigallocatechin-3-gallate	73	11,579.29	0.45
MOL000422	kaempferol	39	3007.39	0.40
MOL000358	beta-sitosterol	27	3520.40	0.38
MOL000354	isorhamnetin	26	1176.60	0.36
MOL000392	formononetin	23	2206.78	0.37
MOL000449	Stigmasterol	23	2619.81	0.36
MOL002773	beta-carotene	19	1344.66	0.36
MOL011072	Quinicine	14	1086.41	0.35
MOL000492	(+)-catechin	4	365.78	0.34
MOL011074	Scillaren A_qt	3	363.09	0.24
MOL004350	Ruvoside_qt	1	0.00	0.21
MOL001771	poriferast-5-en-3beta-ol	1	0.00	0.24
MOL011075	Shikodonin	1	0.00	0.21

**Table 3:** Information table of potential target proteins for the treatment of migraines with ginkgo seeds.

Gene	Target Name	Degree	Betweenness	Closeness
*PTGS2*	*Prostaglandin G/H synthase 2*	12	3313.14	0.50
*PTGS1*	*Prostaglandin G/H synthase 1*	9	1057.28	0.41
*PPARG*	*Peroxisome proliferator activated receptor gamma*	8	1018.63	0.44
*ADRB2*	*Beta-2 adrenergic receptor*	6	611.36	0.40
*GABRA1*	*Gamma-aminobutyric acid receptor subunit alpha-1*	6	386.94	0.39
*NOS3*	*Nitric-oxide synthase, endothelial*	6	291.27	0.38
*BCL2*	*Apoptosis regulator Bcl-2*	5	518.97	0.45
*CASP3*	*Caspase-3*	5	518.97	0.45
*CASP8*	*Caspase-8*	5	675.86	0.44
*AKR1B1*	*Aldose reductase*	5	314.05	0.37
*PRSS1*	*Trypsin-1*	5	162.54	0.38
*F2*	*Thrombin*	5	162.54	0.38
*AKT1*	*RAC-alpha serine/threonine-protein kinase*	4	249.45	0.42
*CASP9*	*Caspase-9*	4	416.57	0.44
*MMP1*	*Matrix metalloproteinase-9*	4	249.45	0.42
*PLAU*	*Urokinase-type plasminogen activator*	4	575.35	0.42
*NOS2*	*Nitric oxide synthase, inducible*	4	238.92	0.36
*AR*	*Androgen receptor*	4	129.72	0.38
*DPP4*	*Dipeptidyl peptidase IV*	4	129.72	0.38
*BAX*	*Apoptosis regulator BAX*	4	406.73	0.44
*KCNH2*	*Potassium voltage-gated channel subfamily H member 2*	3	191.49	0.37
*SCN5A*	*Sodium channel protein type 5 subunit alpha*	3	191.49	0.37
*VEGFA*	*Vascular endothelial growth factor A*	3	167.23	0.40
*MMP2*	*72 kDa type IV collagenase*	3	167.23	0.40
*CAV1*	*Caveolin-1*	3	167.23	0.40
*RELA*	*Transcription factor p65*	3	168.00	0.41
*TNF*	*Tumor necrosis factor*	3	168.00	0.41
*STAT1*	*Signal transducer and activator of transcription 1-alpha/beta*	3	168.00	0.41
*NFKBIA*	*NF-kappa-B inhibitor alpha*	3	199.72	0.40
*ODC1*	*Ornithine decarboxylase*	3	199.72	0.40

## Data Availability

The datasets are available from the corresponding author on request.

## Supplementary Materials

The following supporting information can be downloaded at https://www.mdpi.com/article/10.3390/ijms26189225/s1.
