# Supplementary material for: Ginkgo Seed as Medicine–Food Homology for Migraine: Network Pharmacology and Molecular Docking Insights
Source: Int J Mol Sci. 2025 Sep 21;26(18):9225. doi: 10.3390/ijms26189225 (PMC12470726; doi:10.3390/ijms26189225)
Supplement: Supplementary file 1 [file ijms-26-09225-s001.zip › ijms-3806890-supplementary.pdf]

## Figure S1

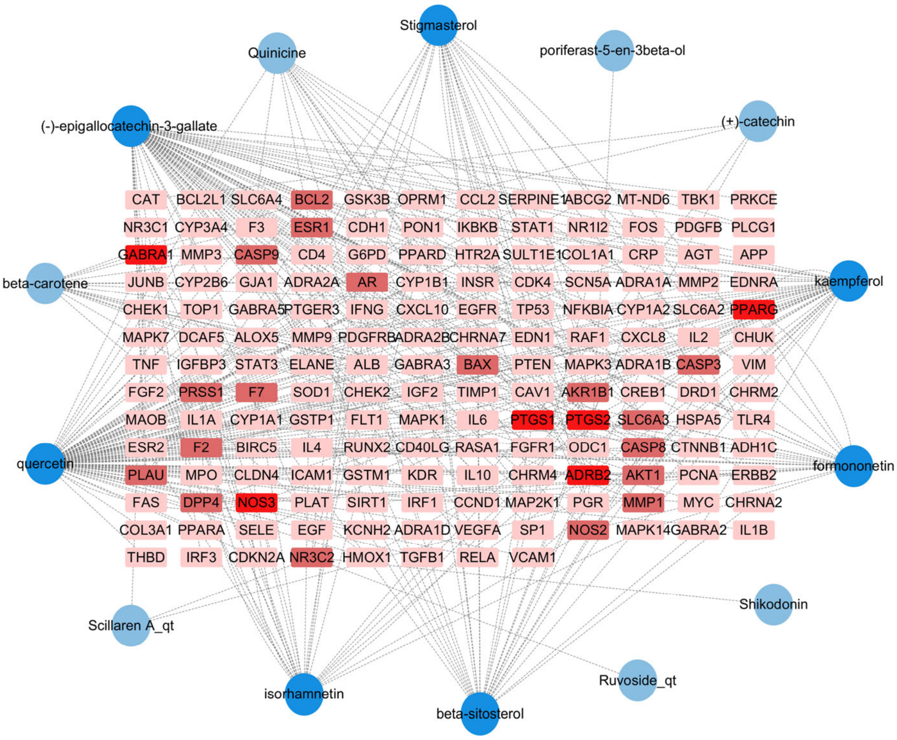


**Figure S1.** The Component-Target protein network. The blue circles represent the 14 candidate active compounds in ginkgo seed. The red rectangles represent the 164 potential hub genes of ginkgo seed against migraine.

## Figure S2

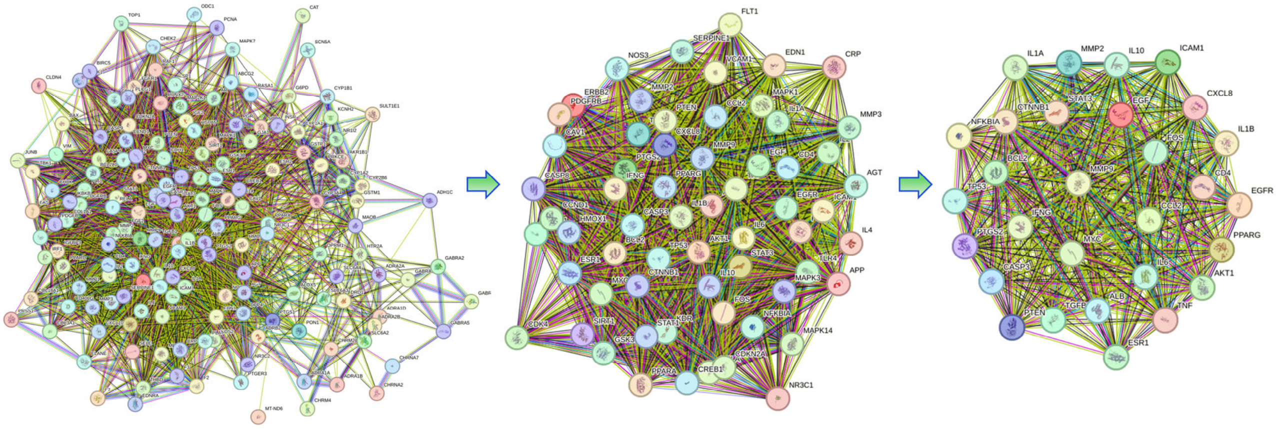


**Figure S2.** Diagrams of protein–protein interaction (PPI) network and the screening process of core target.

**Table S1.** Docking results between target proteins and key pharmacological molecules.

| MOL ID | Molecule Name | PTGS2 | PPARG | TNF | ESR1 | AKT1 | BCL2 | CASP3 |
|---|---|---|---|---|---|---|---|---|
| MOL000358 | beta-sitosterol | -3.97 | -2.49 | -3.17 | -2.97 | -4.02 | -2.1 | -3.47 |
| MOL006821 | (-)-epigallocatechin-3-gallate | -2.54 | -0.1 | -0.64 | 0.65 | 0.12 | 1.13 | -0.61 |
| MOL000392 | formononetin | -4.75 | -2.32 | -3.35 | -3.04 | -3.28 | -2.9 | -3.36 |
| MOL000354 | isorhamnetin | -4.1 | -2.19 | -2.81 | -1.81 | -2.57 | -1.59 | -2.15 |
| MOL000422 | kaempferol | -2.96 | -1.97 | -3.17 | -1.94 | -2.3 | -2.21 | -2.51 |
| MOL000098 | quercetin | -2.0 | -0.78 | -2.48 | -1.36 | -2.0 | -1.97 | -1.75 |
| MOL000449 | stigmasterol | -2.88 | -3.99 | -4.14 | -3.26 | -3.88 | -2.77 | -3.41 |
